# Characteristics of gene mutations in Vietnamese pediatric patients with Inborn Errors of Immunity: a cross-sectional study

**DOI:** 10.1186/s13023-026-04260-2

**Published:** 2026-02-21

**Authors:** Phan Nguyen Lien Anh, Nguyen Minh Tuan, Cao Tran Thu Cuc, Luong Thi Xuan Khanh, Phan Ngo Quang Thach, Nguyen Thanh Hung, Ngo Ngoc Quang Minh, Bui Chi Bao, Huynh Nghia, Hoang Anh Vu, Phan Thi Xinh

**Affiliations:** 1 Department of Hematology, School of Medicine, University of Medicine and Pharmacy at Ho Chi Minh City, Ho Chi Minh City, Vietnam; 2 Department of Hematology, Children’s Hospital 1, Ho Chi Minh City, Vietnam; 3 Blood Transfusion Hematology Hospital, Ho Chi Minh City, Vietnam; 4 University of Health Sciences, Vietnam National University Ho Chi Minh City, Ho Chi Minh City, Vietnam; 5 Center for Molecular Biomedicine, University of Medicine and Pharmacy at Ho Chi Minh City, Ho Chi Minh City, Vietnam; 6 Department of Hematology, School of Medicine, University of Medicine and Pharmacy at Ho Chi Minh City, 217 Hong Bang St, Cho Lon Ward, Ho Chi Minh City, Vietnam

**Keywords:** Gene mutations, Inborn Errors of Immunity, Vietnamese, Pediatric

## Abstract

**Background:**

Inborn Errors of Immunity (IEIs) are rare genetic disorders that cause immunodeficiency. Diagnosis and classification of these disorders are challenging due to the heterogeneous clinical manifestations and genetic mutations. This is the first study to fully describe the spectrum of gene mutations and genetic diversity of IEIs in Vietnamese pediatric patients.

**Methods:**

A cross-sectional study was conducted on pediatric patients diagnosed with IEIs at Children’s Hospital 1, Vietnam, from 2014 to 2024. Ninety-two patients with identified mutations were enrolled. Genetic testing was performed using Sanger, fluorescence in situ hybridization, and whole exome sequencing to identify pathogenic variants. Clinical and epidemiological data were collected from medical records and mutations were classified according to the IUIS 2024 classification.

**Results:**

The study identified 99 mutations in 33 different genes, with X-linked inheritance being predominant (53.2%). The antibody deficiency and immune dysregulation groups were the most common (22.8% for each mutation). Nineteen *novel* mutations were identified, all of which were predictively classified as pathogenic or likely pathogenic. The majority of mutations caused protein truncations, leading to loss of function, especially in the immunodeficiencies affecting cellular and humoral immunity and the phagocyte disorder groups. Clinical manifestations differed markedly between the different gene groups, highlighting the complexity of diagnosing IEIs.

**Discussions:**

The number of cases with mutations causing X-linked inheritance was dominant, which was similar to studies from Asian countries, though different from studies from Africa due to the rates of consanguineous marriage. In addition, the variability between clinical phenotype and genotype in IEIs noted from our study has been a significant obstacle to the classification of diseases and their efficient treatment.

**Conclusions:**

This is the largest single center cohort to date in Vietnam to describe gene mutations in children with IEIs. The results of the study emphasize the importance of genetic testing in the diagnosis and treatment of IEIs, and open possible directions for further research for novel mutations.

**Supplementary Information:**

The online version contains supplementary material available at 10.1186/s13023-026-04260-2.

## Introduction

Inborn Errors of Immunity (IEIs) are a category of rare genetic disorders caused by abnormalities in one or more immune system components, with an incidence of 1/100,000. As a result, IEIs patients are sensitive to infection and are at high risk of severe, protracted, or recurring infections, which can lead to serious complications and death if not being recognized early, or treated appropriately and rapidly. Since the 1990s, the International Union of Immunological Societies (IUIS) has begun to identify and publish inborn immune system abnormalities. This classification is continuously updated and refined to enhance its clinical utility. The IUIS 2024 [1] classification includes 508 distinct IEIs, with an addition of 78 newly identified genes compared to the IUIS 2019 [2] version. The majority of IEIs are single-gene disorders with Mendelian inheritance patterns. Certain types of IEIs are more prominent in specific geographical areas as a result of consanguineous marriages. The majority of classic IEIs are identified with clinical immunological symptoms, followed by molecular diagnosis. However, many immune-related gene mutations result in different phenotypic expressions, making clinical and immunological diagnostic criteria less precise. The same gene may lead to various phenotypes, or the same disease can be caused by multiple genes. The variable molecular characteristics and clinical phenotypes of IEIs make it difficult to approach, to diagnosis accurately and to classify properly, thereby affecting the quality of treatment and prognosis. Children’s Hospital 1 is a tertiary hospital in Vietnam that receives and treats cases diagnosed with IEIs with diverse clinical conditions. Therefore, this study was conducted to clarify the characteristics of gene mutations in pediatric patients with IEIs in Vietnam.

## Methods

### Patients

Patients who were under the age of 16, diagnosed and treated for IEIs at Children’s Hospital 1 between 2014 and 2024, and having pathogenic or likely pathogenic gene mutations or variations were included in this study. The study was approved by the medical ethics committee at Children’s Hospital 1 (No. 154/QD-BVND1). The research participants’ parents/guardians provided written consent. IEIs are classified according to IUIS 2024 classification [1]. A family history of IEIs was described as having parents or siblings with IEIs, or having siblings who died before the age of two as a result of infection. The month of age when the first infection occurred is referred to as the time of beginning of infection.

### Genetic testing

Male patients who presented with typical clinical features of X-linked agammaglobulinemia (XLA), including recurrent bacterial infections at least two times or for the first time but requiring antibiotics combination therapy for more than two weeks, and a complete lack of plasma immunoglobulin A (IgA), IgG, IgM, B lymphocyte count below 1% in peripheral blood, were examined for *BTK* gene mutations using the Sanger sequencing method. Male patients with typical clinical symptoms of Wiskott-Aldrich syndrome, such as recurrent bacterial or viral infections, thrombocytopenia, small platelet size, and atopic dermatitis, were screened for *WAS* gene mutations using the Sanger sequencing technique. Furthermore, cases who were diagnosed with DiGeorge syndrome based on congenital heart defects, thymic aplasia or hypoplasia detected via ultrasound or computed tomography (CT), persistent hypocalcemia, parathyroid insufficiency, and recurrent bacterial, viral, or fungal infections were analyzed for deletions in the *N25*,* TBX1*,* TUPLE1*, and 22q13.3 genes using the CytoCell DiGeorge probe (Cytocell, United Kingdom) with the fluorescent in situ hybridization (FISH) technique.

Clinical cases that were atypical or fit in one of the three categories described above but did not have proper pathogenic mutations were studied further utilizing whole exome sequencing (WES) method.

DNA was extracted and transmitted to Invitae Corporation, USA before January 2023, and to Macrogen between January 2023 and December 2024, for WES analysis with the IEI Panel. This panel contained 474 genes related to IEI. Patients’ samples were tested for mutations in exons and exon-intron junctions, with a high analytical sensitivity (> 99%) for single nucleotide variants (SNVs), minor insertions/deletions (< 15 bp), and exon-level deletions/duplications. The bioinformatics pipeline was used to identify genetic variants, and the WES data was analyzed using a bioinformatics strategy [3]. Only variations with high confidence were categorized and reported using the American College of Medical Genetics and Genomics (ACMG) criteria [4]. Cases with identified or suspected pathogenic mutations/variants were included in the study sample and classified using the IUIS 2024 classification [1]. In certain cases, the presence of the patient’s mutations in his parents or his mother was confirmed using the Sanger sequencing technology.

## Results

### Patients

During the study period, 156 clinically suspected IEI patients were evaluated. It was noted that 92 out of 156 patients carried pathogenic or likely pathogenic variants (58.97%). Among 92 cases carrying mutations, there are 74 males and 18 females belonging to nine IEIs subtypes according to IUIS 2024 classification (Table 1). No cases of IEI phenocopies were found. The immune dysregulation and antibody deficiency group had the most cases (22.8%), followed by the combined immunodeficiencies with associated or syndromic features group (18.5%). Males were dominant in all categories. In 47.8% of cases, the infection occurred before the age of six months. 14.1% of patients had an IEIs family history, including two patients were siblings with osteopetrosis disorders classified as the defects in intrinsic and immunity subtype. The groups with the largest proportion of IEIs family history were defects in the intrinsic and innate immunity group (66.7%) and the phagocyte disorders group (37.5%) (Table 1).

**Table 1:** Patient characteristics

IEIs group	Boy (*n*,% )	Infection before 6 month-old (*n*,% )	Family history of IEIs (*n*,% )	Total cases (*n*)
Immunodeficiencies affecting cellular and humoral immunity	7 (70.0)	8 (80.0)	1 (10.0)	**10**
Combined immunodeficiencies with associated or syndromic features	14 (88.2)	10 (64.7)	1 (11.7)	**17**
Predominantly antibody deficiencies	19 (90.5)	6 (28.6)	1 (4.8)	**21**
Diseases of immune dysregulation	14 (66.7)	4 (19.1)	1 (9.5)	**21**
Congenital defects of phagocyte number or function	8 (81.8)	8 (81.8)	4 (37.5)	**11**
Defects in intrinsic and immunity	4 (66.7)	3 (50.0)	4 (66.7)	**6**
Autoinflammatory disorders	4 (100.0)	2 (50.0)	0 (0.0)	**4**
Complement deficiencies	1 (100.0)	1 (100.0)	0 (0.0)	**1**
Bone marrow failure	1 (100.0)	0 (0.0)	0 (0.0)	**1**
Total	73 (80.4)	43 (47.8)	12 (14.1)	**92**

IEIs: Inborn Errors of Immunity

### Genetic test results

99 different variants involving 33 genes were found in 92 cases. With the exception of four cases with mutations in both autosomal dominant (AD) and autosomal recessive (AR) inheritance, 49 cases (53.2%), 31 cases (33.7%), and 8 cases (8.7%) had X-linked (XL), AR, and AD inheritance, respectively. In the bone marrow failure (100%) and antibody deficiencies (90.5%) groups, XL inheritance was the most common. Furthermore, the complement deficiency (100%), immune dysregulation (85.7%), and defects in intrinsic and innate immunity (83.3%) groups were most affected by AR inheritance (Table 2).

**Table 2:** Genetic distribution according to each inborn Erros of Immunity Categories

IEIs group	*N*^o^ of genes	Heredity pattern (*N*^o^ of cases )				*N*^o^ of total variants (*n* = 99)		*N*^o^ of parental segregation analysis variants (*n* = 37) ^#^	
		Total	AR	AD	XL	Total	novel	Findings in parents/mother	De novo
Immunodeficiencies affecting cellular and humoral immunity	5	10	4	0	6	12	0	6	0
Combined immunodeficiencies with associated or syndromic features	3	17	0	5	12	14	3	6	1
Predominantly antibody deficiencies	3	21	0	2	19	21	6	2	2
Diseases of immune dysregulation	10	21	18	0	3	28	7	12	0
Congenital defects of phagocyte number or function	5	11	3	0	8	13	3	6	0
Defects in intrinsic and immunity	4	6	5	1	0	7	0	5	1
Autoinflammatory disorders	1	0	4*	0	0	2	0	0	0
Complement deficiencies	1	1	1	0	0	1	0	0	0
Bone marrow failure	1	1	0	0	1	1	0	0	0
Total	**33**	**92**	**35**	**8**	**49**	**99**	**19**	**37**	**4**

AD: autosomal dominant; AR: autosomal recessive; IEIs: Inborn Errors of Immunity; No: number; XL: X-linked

*: Among the four autoinflammatory cases carrying MEFV variants with an AR inheritance pattern, recent evidence suggests that this gene may also follow an AD inheritance mode (OMIM 608107)

#: Of the 99 variants identified, only 37 underwent parental testing - maternal testing for XL inheritance, and testing of both parents for AD or AR inheritance - to determine whether the variants were de novo

Of the 99 variants, 19 variants (19.2%) were *novel* mutations that had not been published in the literature, most commonly found in the antibody deficiencies (28.6%). The above variants were not found in the immunodeficiencies affecting cellular and humoral immunity, complement deficiencies, and bone marrow failure groups. To identify the *de novo* mutations, 12 XL inheritance variations were examined in the patients’ mothers, four AD inheritance variants, and 25 AR inheritance variants were examined in both parents. All 12 XL inheritance variants and 25 AR inheritance variants were found in the mother or both parents. However, all four AD inheritance variants were *de novo* mutations, but all were published mutations. In the four AD inheritance variants, there were mutations c.476 A > G in the *IKZF1* gene and c.2557 C > T in the *NFKB2* gene, both of which belonged to the antibody deficiencies group; The c.1268G > A mutation in the *STAT3* gene belonged to the combined immunodeficiencies with associated or syndromic features group and the c.1154 C > T mutation in the *STAT1* gene belonged to the defects in intrinsic and immunity group (Table 2).

All 19 variants were examined in accordance with the ACMG categorization and determined to be either pathogenic or possibly pathogenic using the predictive software. Furthermore, certain variants have been published (*CD40L* c.156 + 2T > A [5], *WAS* c.913 C > T, *WAS* c.913 C> T [6]; *LRBA* c.1933 C > T and *LRBA* c.949 C> T [7]) and investigated for protein function (*CD40L* c.156 + 2T > A) by our team (Table 3).

**Table 3:** Details of previously unreported variants

Gene	Variant	Protein	ACMG Classification	ACMG Prediction
*WAS*	c.1148dup	p.P348fsX111	Pathogenic	PVS1
	c.397_402dup	p.E133_A134dup	Likely pathogenic	PM1, PM4, PM2
	c.913 C > T	p.Q305X	Pathogenic	PVS1, PM2, PP3
*BTK*	c.1061 C > T	p.T354I	Likely pathogenic	PVS1, PM2
	c.1027 C > T	p.Q343X	Pathogenic	PVS1, PM2, PP3
	c.1608_1609insA	p.V537fsX3	Likely pathogenic	PVS1, PM2
	c. 1898G > T	p.C633F	Likely pathogenic	PVS1, PM2
	c.1382_1383dup	p.G462fsX23	Likely pathogenic	PVS1, PM2
	c.462 C > A	p.C154X	Likely pathogenic	PVS1, PM2
	c.213_214insT	p.N72X	Likely pathogenic	PVS1, PM2
*STXBP2*	c.94 A > T	p.I32F	Likely pathogenic	PVS1, PM2
*LYST*	c.6122_11267	p.D2041fsX20	Likely pathogenic	PVS1, PM2
*RAB27A*	c.244 C > A	p.T75K	Likely pathogenic	PM5, PM2
*LRBA*	c.1933 C > T	p.R465X	Pathogenic	PVS1, PM2, PP3
	c.949 C > T	p.R317X	Pathogenic	PVS1, PM2, PP3
*AIRE*	c.473delT	p.L158fsX220	Likely pathogenic	PVS1, PM2
*CYBB*	c.1390 C > T	p.Q464X	Pathogenic	PVS1, PM2, PP3
	c.381delG	p.N128fsX12	Likely pathogenic	PVS1, PM2
*MPO*	c.1805G > A	p.W602X	Pathogenic	PVS1, PM2, PP3

ACMG: American College of Medical Genetics and Genomics; PVS: pathogenic very strong; PM: pathogenic moderate; PP: pathogenic supporting

Regarding the effect of mutations on the proteins, 48 variants (48.5%) including 1 inframe and 47 missense mutations were reading frame conservation, while the remaining variants belonged to the large deletion, splicing, frameshift, and nonsense mutations (51.5%) (Table 4 and Supplementary Table S1).

**Table 4:** Distribution of mutations by Inborn Errors of Immunity categories

IEIS Group	Large Deletion	Splicing	Frameshift	Nonsense	In-Frame	Missense	Total
Immunodeficiencies affecting cellular and humoral immunity	0	3	0	3	1	5	12
Combined immunodeficiencies with associated or syndromic features	1	0	3	2	0	8	14
Predominantly antibody deficiencies	2	0	2	5	0	12	23
Diseases of immune dysregulation	1	2	7	5	0	13	28
Congenital defects of phagocyte number or function	0	1	3	6	0	3	13
Defects in intrinsic and immunity	0	0	3	1	0	3	7
Autoinflammatory disorders	0	0	0		0	2	2
Complement deficiencies	0	0	0	1	0	0	1
Bone marrow failure	0	0	0		0	1	1
Total	**4**	**6**	**18**	**23**	**1**	**47**	**99**

IEIs: Inborn Errors of Immunity

Twelve mutations were detected in 10 cases of the immunodeficiencies affecting cellular and humoral immunity group, including three cases with *CD40L* mutations causing CD40 ligand deficiency and seven cases with the severe combine immunodeficiency (SCID). In the SCID group, two cases with *ADA* mutations belonged to SCID T-B-NK+; three cases with *IL2RG* mutations and one case with *JAK3* mutations belonged to SCID T-B + NK-; and one case with *RAG1* mutations belonged to SCID T-B-NK+. No case in the SCID T-B + NK+ group was detected. The combined immunodeficiencies with associated or syndromic features group consisted of 17 cases with 11 well-known mutations and 3 *novel* variants. Particularly, 11 cases had a mutation in the *WAS* gene, which caused Wiskott Aldrich syndrome; two patients had a mutation in the *STAT3* gene, which caused hyper-IgE syndrome; and four cases had a deletion in the chromosome 22q11, which caused DiGeorge syndrome. This group’s three *novel* variations are all related to Wiskott Aldrich syndrome. Of the 21 patients in the antibody deficiency group, seven *novel* variations and 14 well-known mutations were found. Two of these 21 cases had AD inheritance, including one with *IKZF1* deficiency and one with *NFKB1* deficiency, both of which were *de novo* variants and not present in the parents. Nineteen of these individuals had XL inheritance, having *BTK* mutations that caused XLA syndrome (Supplementary Table S1).

There were 22 well-known mutations and 7 *novel* variants detected in 21 patients with the immune dysregulation group. The familial hemophagocytic lymphohistiocytosis (FHL) group included one case of FHL2, six cases of FHL3, and five cases of FHL5. Among them, two patients carried the c.965_967 > 68 bp mutation in the *UNC13D* gene and two patients carried the c.58 C > T mutation in the *STXBP2* gene, and these patients were not related by consanguinity. There were three cases in the HLH group with hypopigmentation carrying *LYST*, *RAB27A1* and *AP3B1* mutations causing Chediak Higashi syndrome, Griscelli type 2 syndrome and Hemansky Puddle 2 syndrome, respectively. The remaining 5 cases included 1 case carrying an *AIRE* mutation causing autoimmune polyendocrinopathy-candidiasis-ectodermal dystrophy (APECED) syndrome and two cases of LRBA deficiency, 1 case with an *SH2D1A* mutation causing X-linked lymphoproliferative (XLP) disorder and 1 case with a *FOXP3* mutation causing APECED. The XLP and APECED groups are the two groups not containing new variants in the immune dysregulation group.

There were 10 well-known mutations and 3 *novel* variants found in 11 cases of defects of phagocyte number or function group including 4 cases with neutropenia and 7 cases with reduced neutrophil function. There were 3 cases with *ELANE* mutations causing severe congenital neutropenia (SCN) and 1 case with *SBDS* mutation causing Swatchman Diamond syndrome. In addition, there were 5 cases with *CYBB* mutations causing chronic granulomatous disease (CGD) (3 mutations and 2 *novel* variants), 1 case with *ITGB2* mutation causing leukocyte adhesion deficiency type 1 (LAD1) and 1 case with *MPO* variant causing MPO deficiency all in the group with reduced neutrophil function.

Of six cases in the defects in intrinsic and innate immunity group, there was one case of NBAS deficiency due to *NBAS* mutation, one case with *STAT1* mutation causing chronic mucocutaneous candidiasis (CMC) and one case with *IL12RB1* mutation causing Mendelian susceptibility to mycobacterial disease (MSMD). Notably, this group had three cases with *TCIRG1* mutation causing osteopetrosis disease, of which two thirds of the cases were siblings.

In the autoinflammatory group, there were 4 cases with *MEFV* mutation causing Familial Mediterranean fever (FMF). The complement deficiency group had one case with C9 deficiency and the bone marrow failure group had one case with *DKC1* mutation causing Dyskeratosis congenita (Supplementary Table S1).

Treatment regimens varied among groups. Hematopoietic stem cell transplantation was not performed at our hospital during the study period, and intravenous immunoglobulin has been used for prophylactic treatment of infections in predominent antibody deficiency patients since 2020. The median follow-up time was 20.5 months (range: 1–158 months). The overall mortality rate was 26.1%, with the highest mortality observed in the group with immunodeficiencies affecting both cellular and humoral immunity, reaching up to 50% (Supplementary Table S2).

## Discussion

Our study is the first study in Vietnam to fully describe the mutation distribution of IEIs groups. The antibody deficiency (22.8%), immune dysregulation (22.8%), and combined immunodeficiencies with associated or syndromic features (18.5%) groups were predominant (Table 1), similar to the results of Mohammadzadeh [8], however, the results of Krishna [9] and Al-Hert [10] showed that the immunodeficiencies affecting cellular and humoral immunity occurred at a high rate. This difference may be due to differences in sample size, race, and culture. In the study of Al-Hert [10], cases that had not been genetically analyzed and were not classified according to IUIS 2024 were included.

In 92 cases, genetic analysis identified 99 variants related to 33 genes causing IEIs. The number of cases with mutations causing XL inheritance was dominant (53.2%) (Table 2), similar to studies from Asian countries [11–14], but different from studies by Al-Herz [15] in Kuwait and Al-Mousa [16] in Africa, in which AR inheritance was dominant in 90% and 60% of patients, respectively. In countries with high rates of consanguineous marriage, such as Kuwait (54%), AR inheritance was dominant [15]; while in countries with low rates of consanguineous marriage, XL inheritance was dominant, which were similar to our study.

Of the 33 mutated genes found in this study, only *BTK*, *WAS* genes and 22q11 deletion were detected using the classic Sanger and FISH techniques. The remaining 30 genes were detected using next generation sequencing (NGS) technique (Supplementary Table S1). The advent of NGS has allowed the analysis of more genes at the same time, significantly increasing the ability to detect mutations and better understand of the genetic basis of IEIs [17, 18].

There were 19 (19.2%) *novel* variants found in this study (Table 3), lower than the Thai study (30%) [19]. The difference may due to the genetic analysis methods, in the Thai study all cases were analyzed by WES, and the study was published before 2022. The IEIs disease group has recently received attention, so the ability to detect *novel* mutations has also decreased over time. Of the 19 novel variants, up to 14 variants caused early frameshifting, severely affecting protein function. Therefore, the ACMG pathogenicity prediction score was extremely high, with 7 pathogenic variants and 12 likely pathogenic variants, highlighting great potential for further research to clarify the pathogenic mechanisms and expand knowledge about the molecular pathogenesis of IEIs.

There were 51 (51.5%) large deletion, slicing, frameshift and nonsense mutations (Table 4), higher than that of reported by Al-Herz (43%) [15]. Protein truncating variants were highly prevalent in complement deficiency, defects of phagocyte number or function, immunodeficiencies affecting cellular and humoral immunity and immune dysregulation groups, which are groups with severe clinical manifestations (Supplementary Table S1), suggesting a genotype-phenotype correlation in IEIs.

The variability between clinical phenotype and genotype in IEIs is one of its noteworthy characteristics; this has long been a significant obstacle to the classification of diseases and their efficient treatment. Numerous distinct phenotypes can be induced by the same gene, and a disease can be brought on by a variety of gene mutations [1, 2]. This makes the process of classifying the disease using clinical and immunological criteria more difficult. We found that the SCID group had mutations in many different genes, leading to different phenotypes. These included *ADA*,* IL2RG*,* JAK3*, and *RAG1* mutations, which were seen in SCID T-B-NK-, T-B + NK-, and SCID T-B-NK+ phenotypes, respectively.

In addition, in FMF caused by the *MEFV* gene (OMIM 608107), there are 2 inheritance types AR and AD. Patients in the AD group presented with milder clinical manifestations and better treatment response compared with those in the AR group [20]; the clinical severity varies depending on the mutation [21]. In our study, one case with homozygous mutation developed severe complications on the kidneys and hearing, whereas two cases with heterozygous mutations did not presented with the above complications, despite all patients carrying the same mutation (c.442G > C) on the *MEFV* gene, similar to the report of Talaat [20].

In the AR inheritance group, mutations in two alleles usually cause the disease. However, in our study, this group included 7 cases with only one allele mutation, including 2 cases with *UNC13D* mutations, 2 cases with *STXBP2* mutations, 1 case with *MPO* mutations, 1 case with *IL12RB1* mutations, and 1 case with *C9* mutations. All of them met the typical clinical and biological diagnostic criteria. There is increasing evidence that heterozygous variants may also contribute to the clinical manifestations because the remaining variant may not be identified with WES or due to environmental inluences [22, 23]. To achieve an accurate diagnosis, it is therefore required to integrate clinical, pedigree, immunological function testing, and gene mutations employing WES/NGS, even microarray, CNVs [24]. The results may provide new treatment opportunities, leading to improved patient outcomes and enabling suitable genetic counseling.

In our study, the group with immunodeficiencies affecting cellular and humoral immunity exhibited the highest mortality rate, likely due to the unavailability of hematopoietic stem cell transplantation during the study period. Mortality was also relatively high in the predominant antibody deficient group, as prophylactic intravenous immunoglobulin was not widely available in Vietnam before 2020. In contrast, all patients in four groups including congenital defects of phagocyte number or function, autoinflammatory disorders, complement deficiencies, and bone marrow failure survived at the end of the study.

Although this study showed unique results regarding the diagnosis and treatment of Vietnamsese IEIs patients, there are some limitations. These include the single-center study design and the lack of functional validation for 18 of the 19 novel variants, despite their high predictive value for disease according to ACMG criteria. Additionally, in some cases of inherited AR disease, only one heterozygous variant was detected. These limitations provide potential directions for our further studies.

## Conclusion

To date, this study is the largest single center cohort to describe the characteristics of gene mutations in 92 children with IEIs in Vietnam. The detection of gene mutations helps to accurately diagnose the group of IEIs, resulting in suitable treatment and genetic counseling.

## Supplementary Information

Below is the link to the electronic supplementary material.

Supplementary Material 1

## Data Availability

The datasets used during the current study are available from the corresponding authors on reasonable request.
