# Supplementary material for: Characteristics of gene mutations in Vietnamese pediatric patients with Inborn Errors of Immunity: a cross-sectional study
Source: Orphanet J Rare Dis. 2026 Feb 21;21:112. doi: 10.1186/s13023-026-04260-2 (PMC13032614; doi:10.1186/s13023-026-04260-2)
Supplement: Supplementary file 1 — Supplementary Material 1 [file 13023_2026_4260_MOESM1_ESM.docx]

**Supplementary Table S1. Details of Mutations by Inborn Errors of Immunity Groups**

| **IEIS Group** | **Gene/ Chromosomal region** | **Heredity** | **Number of cases** | **DNA change** | **Protein** | |
| --- | --- | --- | --- | --- | --- | --- |
| Immunodeficiencies affecting cellular and humoral immunity (n = 10); 12 variants | | | | | | |
| CD40 ligand deficiency | *CD40L* | XL | 1 | c.156+2T>A |  | |
|  |  |  | 1 | c.654C>A | p.C218X | |
|  |  |  | 1 | c.433_435del | p.Y146del | |
| SCID T-B-NK- | *ADA* | AR | 1 | c.532delG/c.532delG | p.V178X/ p.V178X | |
|  |  |  | 1 | c.466C>T/c.867C>A | p.R156C/ p.N289K | |
| SCID T-B+NK- | *IL2RG* | XL | 1 | c.454+1G>A |  | |
|  |  |  | 1 | c.930G>A | p.W310X | |
|  |  |  | 1 | c.116-1G>T |  | |
|  | *JAK3* | AR | 1 | c.1763A>C/ c.1763A>C | p.H588P/p.H588P | |
| SCID T-B-NK+ | *RAG1* | AR | 1 | c.2521C >T/ c.1181G>A | p.R841W/p.R394Q | |
| **Combined immunodeficiencies with associated or syndromic features (n= 17); 14 variants** | | | | | |  |
| DiGeorge syndrome | 22q11DS | AD | 4 | 22q11.2del |  |  |
| Hyper IgE syndrome | *STAT3* | AD | 1 | c.1025G>A | p.G342D |  |
|  |  |  | 1 | \| c.1268G>A \| \| --- \| | p.R423Q |  |
| Wiskott Aldrich syndrome | *WAS* | XL | 1 | c.91G>A | p.E31K |  |
|  |  |  | 1 | c.134C>T | p.T45M |  |
|  |  |  | 1 | c.1157del | p. P386fsX59 |  |
|  |  |  | 1 | **c.1148dup** | **p.P348fsX111** |  |
|  |  |  | 1 | **c.397_402dup** | **p.E133_A134dup** |  |
|  |  |  | 1 | c.298_312del | p.E100_Q104del |  |
|  |  |  | 1 | **c.913C>T** | **p.Q305X** |  |
|  |  |  | 1 | c.847G>A | p.D283N |  |
|  |  |  | 1 | c.631C>T | p.R211X |  |
|  |  |  | 1 | c.404A>C | p.Q135P |  |
|  |  |  | 1 | c.1221del | p.N408fsX37 |  |
| **Predominantly Antibody Deficiencies (n = 21); 21 variants** | | | | | | |
| X-linked agammaglobulinemia | *BTK* | XL | 1 | c.862C>T | p.R288W | |
|  |  |  | 1 | **c.1061C>T** | **p.T354I** | |
|  |  |  | 1 | c.1205T>C | p.L402P | |
|  |  |  | 1 | c.1567_1631del | p.A523fsX527 | |
|  |  |  | 1 | **c.1027C>T** | **p.Q343X** | |
|  |  |  | 1 | c.1855C>A | p.P619T | |
|  |  |  | 1 | c.763C>T | p.R255X | |
|  |  |  | 1 | c.1745C>A | p.A582D | |
|  |  |  | 1 | **c.1608_1609insA** | **p.V537fsX3** | |
|  |  |  | 1 | **c. 1898G>T** | **p.C633F** | |
|  |  |  | 1 | c.1489C>T | p.Q497X | |
|  |  |  | 1 | c.1921C>T | p.R641C | |
|  |  |  | 1 | **c.1382_1383dup** | **p.G462fsX23** | |
|  |  |  | 1 | c.1696C>T | p.P566S | |
|  |  |  | 1 | **c.462C>A** | **p.C154X** | |
|  |  |  | 1 | c.1030T>G | p.Y344D | |
|  |  |  | **1** | **c.213_214insT** | **p.N72X** | |
|  |  |  | 1 | c.1457T>G | p.L486R | |
|  |  |  | 1 | c.1037T>A | p.L346Q | |
| IKZF1 deficiency | *IKZF1* | AD | 1 | c.476A>G | p.N159S | |
| NFKB2 deficiency | *NFKB2* | AD | 1 | c.2557C>T | p.R853X | |
| **Diseases of Immune Dysregulation (n = 21); 29 variants** | | | | | | |
| FHL type 2 | *PRF1* | AR | 1 | c.98G>A/c.10C>T | p.R33H/p.R4C | |
| FHL type 3 | *UNC13D* | AR | 2 | c.3151G>A | p.F985fsX14 | |
|  |  |  | 1 | c.2831-13G>A/c.2831-13G>A |  | |
|  |  |  | 1 | c.965_967>68bp/ c.965_967>68bp | p.A318X/p.A318X | |
|  |  |  | 1 | c.965_967>68bp /c.3151G>A | p.A318X/ p.F985fsX14 | |
|  |  |  | 1 | c.755_756dup/c.1283T>G | p.L253fsX76/ p.L428R | |
| FHL type 5 | *STXBP2* | AR | 1 | c.1463C>T | p.P488L | |
|  |  |  | 1 | c.1430C>T | p.P477L | |
|  |  |  | 1 | c.58C>T/c.37+5G>A | p.R20W | |
|  |  |  | 1 | c.58C>T/**c.94A>T** | p.R20W/**p.I32F** | |
|  |  |  | 1 | c.37+5G>A/c.37+5G>A |  | |
| XLP type 1 | *SH2D1A* | XL | 1 | c.1A>G | p.M1V | |
|  |  |  | 1 | c.163C>T | p.R55X | |
| Chediak Higashi syndrome | *LYST* | AR | 1 | c.9449del /**c.6122_11267 (exon 23- 53 deletion)** | p.N3150fsX35/ **p.D2041fsX20** | |
| Griscelli syndrome type 2 | *RAB27A1* | AR | 1 | **c.244C>A**/c.377delC | **p.T75K** /p.P126fsX3 | |
| HPS type 2 | *AP3B1* | AR | 1 | c.1945C>T/c.1255C>T | p.R649/p.Q419 | |
| LRBA deficiency | *LRBA* | AR | 1 | **c.1933C>T/c.949C>T** | **p.R465X/p.R317X** | |
|  |  |  | 1 | **c.2315T>C/**c.3995T>C | **p.L772P/**p.M1332T | |
| APECED | *AIRE* | AR | 1 | c.769C>T/**c.473delT** | p.R257X/ **p.L158fsX220** | |
| IPEX | *FOXP3* | XL | 1 | c.227del | p.L76fsX53 | |
| **Congenital defects of phagocyte number or function (n= 11); 13 variants** | | | | | |  |
| Severe congenital neutropenia | *ELANE* | XL | 1 | c.640G>A | p.G214R |  |
|  |  |  | 1 | c.215T>G | p.V72G |  |
|  |  |  | 1 | c.176T>G | p.L59R |  |
| Shwachman Diamond syndrome | *SBDS* | AR | 1 | c.184A>T / c.297_300delAAGA | p.K62X / p.E99fsX21 |  |
| LAD type 1 | *ITGB2* | AR | 1 | c.1030G>T/c.59-1G>A | p.E344X |  |
| Chronic granulomatous disease | *CYBB* | XL | 1 | **c.1390C>T** | **p.Q464X** |  |
|  |  |  | 1 | c.75del | p.F26fsX35 |  |
|  |  |  | 1 | **c.381delG** | **p.N128fsX12** |  |
|  |  |  | 1 | c.1133A>G | D378G |  |
|  |  |  | 1 | c.965G>A | p.G322E |  |
| MPO deficiency | MPO | AR | 1 | **c.G1805A** | **p.W602X** |  |
| **Defects in Intrinsic and Immunity (n =6); 7 variants** | | | | | |  |
| NBAS deficiency | *NBAS* | AR | 1 | c.586C>T/c.6859G>T | p.Q196X/p.D2287Y |  |
| Osteopetrosis | *TCIRG1* | AR | 1 | c.238delC/c.1305+2T >C | p.P81fsX84 |  |
|  |  |  | 2 | c.242del/ c.242del | p.P81fsX85/ p.P81fsX85 |  |
| Chronic Mucocutaneous Candidiasis | *STAT1* | AD | 1 | c.1154C>T | p.T385M |  |
| MSMD | *IL12RB1* | AR | 1 | c.632G>C | p.R211P |  |
| **Autoinflammatory Disorders (n=4); 2 variants** | | | | | |  |
| Familial Mediterranean fever | *MEFV* | AR | 1 | c.442G>C/ c.442G>C | p.E148Q/p.E148Q |  |
|  |  |  | 2 | c.442G>C | p.E148Q |  |
|  |  |  | 1 | c.2282G>A | p.R761H |  |
| **Complement Deficiencies (n=1); 1 variants** | | | | | |  |
| Complement 9 deficiency | *C9* | AR | 1 | c.346C>T | p.R116X |  |
| **Bone marrow failure (n=1); 1 variants** | | | | | |  |
| Dyskeratosis congenita | *DKC1* | XL | 1 | c.146C>T | p.T49M |  |

**Bold:** *novel* mutation;

XL: X-linked; AR: autosomal recessive; AD: autosomal dominant; SCID: severe combine immunodeficiency; FHL: Familial hemophagocytic lymphohistiocytosis; XLP: X-linked lymphoproliferative; HSP: Hermansky-Pudlak syndrome; APECED: autoimmune polyendocrinopathy candidiasis ectodermal dystrophy; IPEX: Immune Dysregulation Polyendocrinopathy Enteropathy X-linked Syndrome; LAD: leukocyte adhesion deficiency; MSMD: Mendelian susceptibility to mycobacterial diseases;

**Supplementary Table S2: Mortality rates by IEI subgroup**

| **IEIs group** | **Total cases**  **(n)** | **Death**  **(n)** | **Death**  **(%)** |
| --- | --- | --- | --- |
| **Immunodeficiencies affecting cellular and humoral immunity** | 10 | 5 | 50 |
| **Combined immunodeficiencies with associated or syndromic features** | 17 | 3 | 17.6 |
| **Predominantly antibody deficiencies** | 21 | 7 | 33.3 |
| **Diseases of immune dysregulation** | 21 | 8 | 38 |
| **Congenital defects of phagocyte number or function** | 11 | 0 | 0 |
| **Defects in intrinsic and immunity** | 6 | 1 | 16.7 |
| **Autoinflammatory disorders** | 4 | 0 | 0 |
| **Complement deficiencies** | 1 | 0 | 0 |
| **Bone marrow failure** | 1 | 0 | 0 |
| **Total** | **92** | **24** | **26.1** |

IEI: inborn errors of immunity
